# Community-Based Services to Improve Testing and Linkage to Care Among Non–U.S.-Born Persons with Chronic Hepatitis B Virus Infection — Three U.S. Programs, October 2014–September 2017

**DOI:** 10.15585/mmwr.mm6719a2

**Published:** 2018-05-18

**Authors:** Aaron M. Harris, Ruth Link-Gelles, Karen Kim, Edwin Chandrasekar, Su Wang, Nicole Bannister, Perry Pong, Eric Chak, Moon S. Chen, Christopher Bowlus, Noele P. Nelson

**Affiliations:** ^1^Division of Viral Hepatitis, CDC; ^2^Asian Health Coalition, Chicago, Illinois; ^3^Saint Barnabas Medical Center, Center for Asian Health, Livingston, New Jersey; ^4^Charles B. Wang Community Health Center, New York, New York; ^5^Davis School of Medicine, University of California, Sacramento, California; ^6^United States Public Health Service, Rockville, Maryland.

Among an estimated 850,000 to 2.2 million persons with chronic hepatitis B virus (HBV) infection in the United States, 70% are non–U.S.-born (*1*,*2*). All patients require linkage to care, and approximately 20%–40% require antiviral treatment (*3*). Without treatment, one in four persons chronically infected with HBV will die prematurely from liver failure, liver cirrhosis, or hepatocellular carcinoma (*4*). To mitigate morbidity and mortality, CDC funded a cooperative agreement to develop hepatitis B testing and linkage-to-care programs serving non–U.S.-born persons during October 2014–September 2017. This report describes each program’s operational services and partnerships with primary care centers, community-based organizations, and public health departments to recruit non–U.S.-born persons for HBV testing using the hepatitis B surface antigen (HBsAg) and link those whose test results were positive to HBV-directed care (medical visit attendance with monitoring of HBV DNA and liver enzyme tests). Among 10,152 program participants, 757 (7.5%) were HBsAg-positive, indicative of chronic HBV infection; among these, 643 (85%) attended ≥1 medical visit, 587 (78%) received HBV-directed care, and 137 (18%) were prescribed antiviral treatment. Among 273 household contacts of HBsAg-positive persons, 39 (14%) had positive test results for HBsAg. Prevalence of current HBV infection was high in this non–U.S.-born population and among household and sexual contacts of HBV-infected persons. HBV testing and linkage to care can be achieved through partnerships with community organizations, health centers, and public health departments.

HBV testing and linkage-to-care programs serving non–U.S.-born populations were located at a nongovernmental agency in Chicago, Illinois; community health centers in Livingston, New Jersey and New York City, New York; and an academic health center in Sacramento, California. Programs partnered with community-based organizations (CBOs), medical clinics (federally qualified health centers, primary care/specialists) and public health departments to implement various community-based services (Supplementary Table; https://stacks.cdc.gov/view/cdc/53787). Programs performed HBV screening by testing for HBsAg; persons whose test results were positive received a second HBsAg test 6 months later to confirm chronic HBV infection. Testing for antibody (total or immunoglobulin G [IgG]) to hepatitis B core antigen (anti-HBc) and antibody to hepatitis B surface antigen (anti-HBs) was performed when feasible. Linkage to care was defined as documentation of at least one medical visit. Community-based services used to facilitate linkage to care were qualitatively assessed and included community screening events, clinical decision support tools in the electronic medical record (EMR), including flagging of charts of patients who were potentially at high risk for infection, provider education and feedback, and patient navigation, which was defined as individualized efforts to assist patients in accessing health care services (*5*).

Two programs (Chicago and Livingston/New York City) participated in HBV testing and vaccination of household contacts of persons who tested positive for HBsAg. These household contacts were offered HBV testing, and those whose test results were HBsAG-positive were linked to care; persons whose test results were HBsAG-negative with positive anti-HBs results (indicative of immunity through immunization or resolved infection) were reassured; and persons whose test results were negative for all three HBV seromarkers (susceptible to infection) were offered hepatitis B vaccination (3-dose series over 6 months). 

Among persons whose test results were positive for HBsAg, demographic characteristics, including sex, year of birth, race/ethnicity, health insurance status, and country of birth were collected, as were HBV-specific linkage-to-care indicators, including documentation of two medical visits (retention in care), and HBV-directed care, including monitoring HBV DNA, alanine aminotransferase, and hepatitis B e antigen (engaged in care). Information on diagnosis of hepatocellular carcinoma and liver cirrhosis was collected. Risk factor data were collected through voluntary questionnaires. A descriptive analysis was performed, and odds ratios with 95% confidence intervals were calculated using logistic regression.

Among 10,152 program participants, 757 (7.5%) had test results that were positive for HBsAg, indicating the presence of chronic HBV infection. The median age of these patients was 40 years (interquartile range 32–53 years); 344 (45%) were female, 602 (80%) were Asian, and 122 (16%) were black (Table). The most frequently reported countries of origin of participants were China (32%), Vietnam (16%), Myanmar (8%), Taiwan (7%), and Laos (3%). Among the 757 persons whose test results were positive for HBsAg, 634 (84%) attended ≥1 medical visit, 587 (78%) received HBV-directed care, 430 (57%) attended ≥2 medical visits, and 137 (18%) were prescribed antiviral therapy (Figure). Among HBsAg-positive participants, 123 (22%) were not linked to care, either because they received care in a different health system or were lost to follow-up. Nine (1.2%) persons received a diagnosis of hepatocellular carcinoma, and liver cirrhosis was diagnosed in 17 (2.2%). Among 8,837 participants who received anti-HBc testing, 2,832 (32%) tested positive, and among 7,421 tested for anti-HBs, 4,284 (58%) tested positive.

**TABLE:** Percentage of HBsAg-positive persons participating in three programs to increase hepatitis B testing and linkage to care are who received HBV-directed care* and treatment, by demographic characteristics — Sacramento, California; Livingston, New Jersey and New York City, New York; and Chicago, Illinois, October 2014–September 2017

Demographic characteristic	HBsAg–positive	Received HBV–directed care*		Prescribed antiviral	
	No. (% of total)	No. (%)	Unadjusted OR (95% CI)	No. (%)	Unadjusted OR (95% CI)
**Total**	757 (100)	587 (78)	N/A	137 (18)	N/A
**Sex**					
Female	344 (45)	276 (80)	Referent	50 (15)	Referent
Male	413 (55)	349 (85)	1.3 (0.9–1.8)	87 (21)	1.6 (1.1–2.4)
**Age group (yrs)**					
<50	518 (68)	423 (82)	Referent	83 (16)	Referent
>50	239 (32)	164 (69)	0.5 (0.3–0.7)	54 (23)	2.0 (1.4–3.0)
**Race**					
White	17 (2)	14 (82)	Referent	2 (12)	Referent
Black/African	122 (16)	83 (68)	0.5 (0.1–1.7)	7 (6)	2.3 (0.5–10.4)
Asian	602 (80)	474 (79)	0.8 (0.2–2.8)	122 (20)	0.5 (0.1–2.4)
Native American/Pacific Islander	3 (0)	3 (100)	—^†^	1 (33)	3.8 (0.2–62.8)
Other/Unknown	13 (2)	13 (100)	—^†^	5 (38)	—^†^
**Health insurance**					
None	121 (16)	68 (56)	0.3 (0.2–0.4)	19 (16)	0.9 (0.5–1.7)
Private	210 (28)	174 (83)	Referent	41 (20)	Referent
Public	334 (44)	289 (87)	1.3 (0.8–2.1)	56 (17)	0.8 (0.5–1.3)
Missing	92 (12)	56 (61)	N/A	21 (23)	N/A
**Primary language**					
English	229 (30)	178 (78)	1.0 (0.7–1.4)	52 (23)	1.4 (0.9–2.0)
Not English (yes to any other language)	520 (69)	406 (78)	Referent	84 (16)	Referent
Missing	8 (1)	3 (38)	N/A	1 (13)	N/A
**Country of origin**					
U.S.-born	16 (2)	13 (81)	1.3 (0.4–4.5)	3 (19)	1.2 (0.3–4.3)
Non–U.S.-born	732 (97)	567 (77)	Referent	133 (18)	Referent
Missing	9 (1)	7 (78)	N/A	1 (11)	N/A
**Liver disease**					
Cirrhosis (vs. no cirrhosis)	18 (2)	17 (94)	1.3 (0.1–9.9)	14 (78)	13.1 (4.2–40.6)
HCC (vs. no HCC)	9 (1)	9 (100)	—^†^	7 (78)	24.6 (3.0–202.4)
Neither cirrhosis nor HCC	469 (62)	439 (94)	N/A	98 (21)	N/A
Missing	264 (35)	128 (48)	N/A	24 (9)	N/A
**Family history**					
Family history of HBV (vs. no family history of HBV)	190 (25)	158 (83)	1.1 (0.7–1.7)	45 (24)	1.7 (1.1–2.7)
Family history of HCC (vs. no family history of HCC)	66 (9)	53 (80)	0.9 (0.5–1.7)	20 (30)	2.2 (1.2–4.0)
No family history of HBV/HCC	364 (48)	303 (83)	N/A	59 (16)	N/A
Missing	182 (24)	111 (61)	N/A	29 (16)	N/A
**Risk factors (other than non–U.S.-born)**					
No risk factors reported	485 (64)	409 (84)	Referent	82 (17)	Referent
At least one risk factor^§^ reported	139 (18)	124 (89)	1.5 (0.9–2.8)	37 (27)	1.8 (1.1–2.7)
No risk factor data available	133 (18)	54 (41)	N/A	18 (14)	N/A

**Abbreviations:** CI = confidence interval; HBsAg = hepatitis B surface antigen; HBV = hepatitis B virus; HCC = hepatocellular carcinoma; N/A = not applicable; OR = odds ratio.

* Received testing for hepatitis B e antigen, hepatitis B virus DNA, and alanine transaminase.

^†^ Insufficient cell size to calculate OR.

^§^ Injection drug use, men who have sex with men, household contact, sexual contact, multiple sex partners, human immunodeficiency virus–positive.

**FIGURE:**
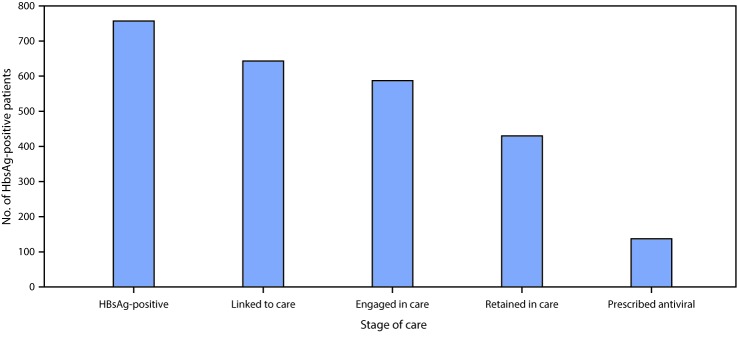
Hepatitis B linkage-to-care continuum* — three U.S. programs, October 2014–September 2017 **Abbreviation:** HBsAg = hepatitis B surface antigen. * Stages in care continuum are the following: linked to care = attended >1 medical visit; engaged in care = received hepatitis B e antigen, hepatitis B virus DNA, and alanine transaminase testing; retained in care = attended >2 medical visits; prescribed antiviral = given treatment with antiviral (approximately 20%–40% of patients with chronic hepatitis B virus infection require treatment).

All three programs implemented community screening events, patient navigation, and EMR strategies. Chicago developed a patient navigation program; Livingston/New York City implemented a provider education and feedback curriculum; and Sacramento used the EMR to flag patients by Asian surname for HBV testing and to link HBsAg-positive patients identified in the EMR to care management to facilitate linkage to care.

HBsAg-positive persons who had health insurance were more likely to receive HBV-directed care than were those who were uninsured (Table). Antiviral treatment was more likely to be prescribed for those HBsAg-positive persons who were men, aged >50 years, who had liver cirrhosis or hepatocellular carcinoma, or who had a family history of HBV infection or hepatocellular carcinoma (Table).

Among 273 household contacts of HBsAg-positive participants, 39 (14%) had positive test results for HBsAg, 83 (30%) had negative test results for HBsAg and positive results for anti-HBc (immune because of resolved infection), and 101 (37%) were anti-HBs-positive and anti-HBc-negative (immune because of vaccination). Fifty (18%) household contacts had negative test results for all three HBV seromarkers, indicating susceptibility; among these persons, 1, 2, and 3 doses of hepatitis B vaccine were received by the end of the project by 37 (74%), 32 (64%), and 25 (50%) participants, respectively.

## Discussion

In this analysis of community-based services used by three HBV testing and linkage-to-care programs that implement CDC’s HBV testing recommendations (*6*) and link persons with chronic HBV infection to care, the overall prevalence of HBV infection among participants was 7.5%. Services used to link 78% of patients to HBV-directed care included community screening events, patient navigation, use of the EMR, and provider education and feedback. A 14% HBV infection rate was observed among household contacts of persons with HBV infection.

HBV testing and linkage to care was accomplished through targeted public health interventions, including partnerships with public health, CBOs, and health centers. Chicago developed partnerships with 11 CBOs, two health centers (a network of federally qualified health centers and a refugee health center), and two public health departments; each site implemented a clinic-based patient navigation program to facilitate access to care for persons with HBV infection. The Livingston/New York City program held HBV testing events in collaboration with community partners, local health departments, and its hospital; the program reported that combining community screening events with general health fairs led to more persons receiving HBV screening. Livingston developed a free HBV testing coupon for testing at the affiliated laboratory; HBsAg-positive patients received care at a primary care center (Livingston) and a federally qualified health center (New York City). Clinics offered trainings in HBV testing and linkage to care, followed by provider feedback to assess performance. Partnerships with local health departments improved HBV case reporting and contact testing. The program in Sacramento implemented community services, including HBV testing events at health fairs and faith-based centers; HBsAg-positive persons with insurance received HBV care within the health system, and those without insurance received care at student-run clinics.

EMR strategies have previously been documented to increase HBV testing rates (*7*); the program in Chicago included EMR prompts to identify populations at high risk, the program in Livingston modified their EMR to include HBV testing order sets (clinical decision support tools that include laboratory tests recommended for evaluating patients with chronic HBV infection), and the program in Sacramento developed an algorithm to flag charts of Asian persons to recommend testing as well as monthly EMR querying of HBsAg-positive patients to identify those not currently in care. A critical linkage-to-care strategy was the use of patient navigators at all sites (*5*,*8*). Patient navigators received training about HBV disease effects, cultural competency, insurance evaluation, the marketplace insurance application process, development of bilingual education materials, and scheduling medical appointments and patients’ guidance through health systems (*5*).

An estimated 20%–40% of HBsAg-positive persons require antiviral treatment (*3*), and this initiative demonstrated that 18% of identified HBsAg-positive patients received antiviral treatment. Reasons for not treating might include cost, access to care, patient preferences, or variation in clinical guidelines used (*9*,*10*).

The 14% HBV infection rate among household contacts in this population is higher than the 0.3% reported in the general population (*2*), highlighting the importance of screening household contacts of persons with HBV infection. Persons who are not immune are at risk for infection, and in these programs, 74% of susceptible household contacts received at least 1 dose of hepatitis B vaccine.

The findings in this report are subject to at least four limitations. First, each program used services specific to their catchment population, which limits generalizability. Second, linkage-to-care indicators might be underestimated because some participants had pending appointments that were not included at the end of the project period. Third, ascertainment of factors associated with progression in the care continuum is limited because of missing data (not all characteristics were documented). Finally, the proportion of treatment-eligible persons determined by clinical guidelines was not assessed.

HBV testing and linkage to care can be achieved among hard-to-reach populations through partnerships with community organizations, health centers, and public health departments. Household and sexual contacts of HBV-infected persons should be tested and linked to care.

**Summary**

**What is already known about this topic?**

Among the 850,000 to 2.2 million U.S. residents with chronic hepatitis B virus (HBV) infection, approximately 70% are non–U.S.-born; nearly two thirds are unaware of their infection status, and <30% are linked to care and treatment.

**What is added by this report?**

CDC funded three programs to develop hepatitis B testing and linkage-to-care programs serving non–U.S.-born persons during 2014–2017; 78% of persons with chronic HBV infection were linked to care using community-based services. HBV infection rate among household contacts of HBsAg-positive persons was 14%.

**What are the implications for public health practice?**

HBV testing and linkage to care can be achieved among hard-to-reach populations through partnerships with community organizations, health centers, and public health departments.
